# Supplementary material for: Pangenome-based human genome analysis improves trait association and genomic prediction
Source: bioRxiv. 2026 Jul 3:2026.07.01.735728. Preprint. [Version 1] doi: 10.64898/2026.07.01.735728 (PMC13345107; doi:10.64898/2026.07.01.735728)
Supplement: Supplement 3 [file NIHPP2026.07.01.735728v1-supplement-3.pdf]

bioRxiv preprint doi: https://doi.org/10.64898/2026.07.01.735728; this version posted July 3, 2026. The copyright holder for this preprint (which was not certified by peer review) is the author/funder, who has granted bioRxiv a license to display the preprint in perpetuity. It is made available under a CC-BY 4.0 International license.

Supplementary Figures

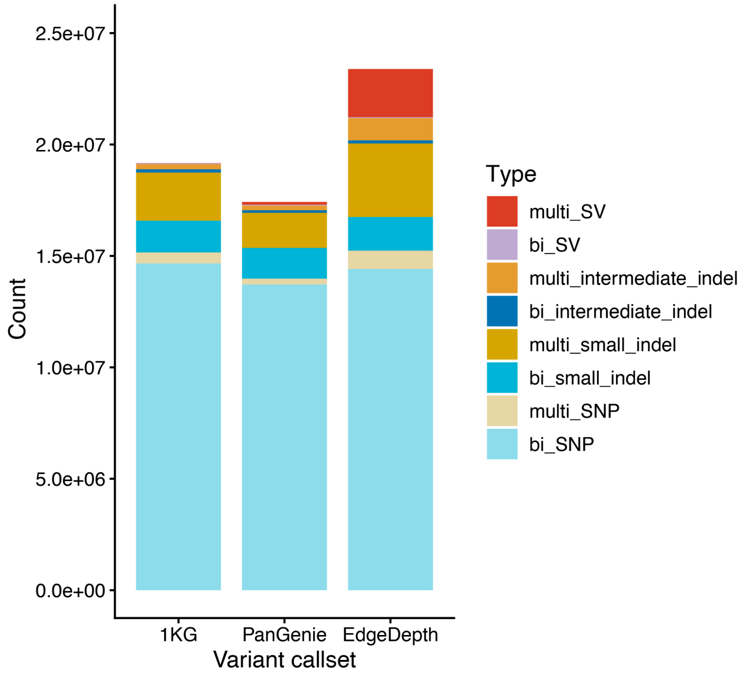

Supplementary Figure 1. Variant type composition in each callset. Variant counts are shown by type after filtering for variant quality and allele frequency, and after rescue of variants matched across callsets (see Methods). Variant types include biallelic or multiallelic variants (bi_ or multi_) and SNPs (1 bp), small indels (2-9 bp), intermediate sized indels (10-49 bp) and SVs (≥50 bp).

bioRxiv preprint doi: https://doi.org/10.64898/2026.07.01.735728; this version posted July 3, 2026. The copyright holder for this preprint (which was not certified by peer review) is the author/funder, who has granted bioRxiv a license to display the preprint in perpetuity. It is made available under a CC-BY 4.0 International license.

a

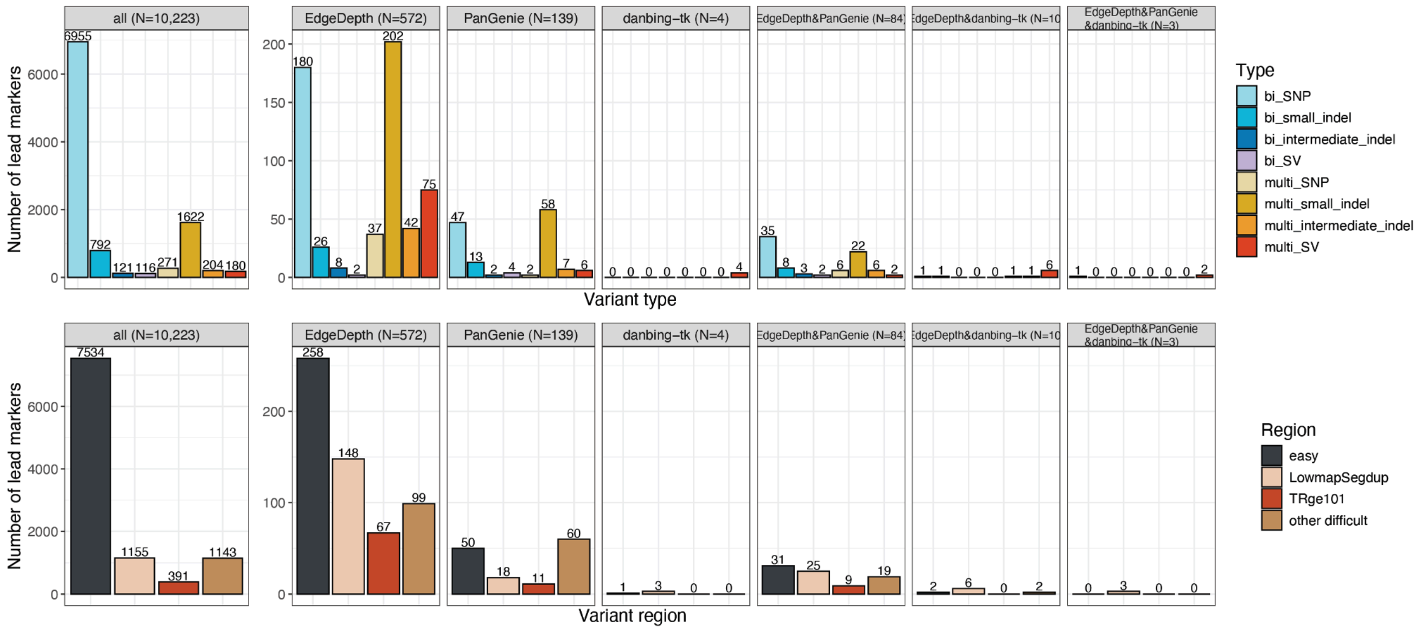

b

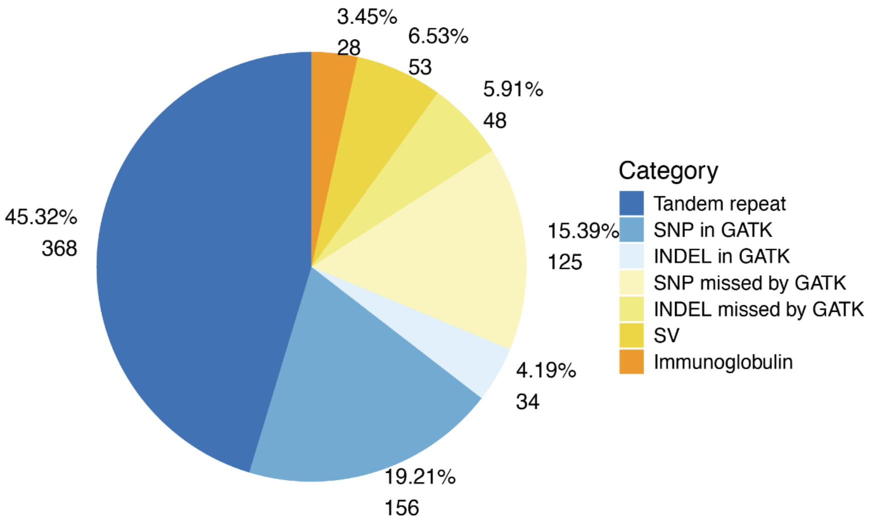

Supplementary Figure 2. The 812 eGenes with a pangenome power boost. Variant type composition of lead eQTL markers and the underlying mechanisms of the 812 eGenes at which pangenome variants improved the eQTL signal, defined as a >20% increase in -log10(nominal p-value) for the most significant variant across all pangenome callsets relative to the most significant 1KG variant. This figure follows the same format as Fig. 3c,d. **(a)** Composition of lead eQTL markers by variant type (top) and genomic region (bottom). The left panel shows all eGenes from the joint analysis (N = 10,223). The right panels show the 812 improved eGenes split by the driving callset, defined by if the callset reported any variant with >20% of improvement. **(b)** Pie chart of the mechanisms underlying the 812 improved lead variants, including tandem repeat, SNP in 1KG, INDEL in 1KG, SNP missed in 1KG, INDEL missed by 1KG, SV, and immunoglobulin.

bioRxiv preprint doi: https://doi.org/10.64898/2026.07.01.735728; this version posted July 3, 2026. The copyright holder for this preprint (which was not certified by peer review) is the author/funder, who has granted bioRxiv a license to display the preprint in perpetuity. It is made available under a CC-BY 4.0 International license.

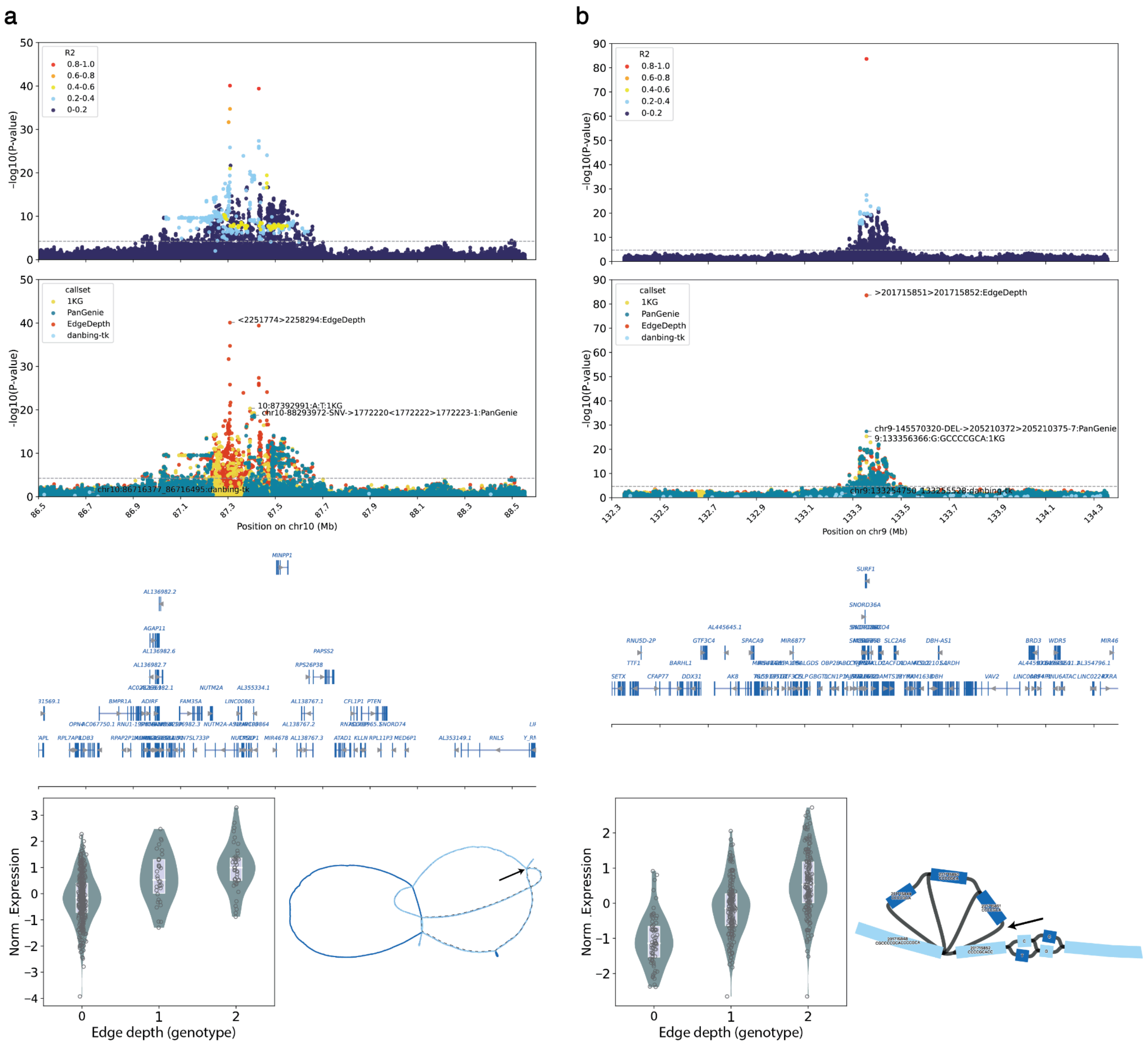

Supplementary Figures 3 and 4. eQTL signals and pangenome graph structures for noteworthy examples. Shown are four representative genes: *MINPP1* (Supplementary Fig. 3a), *SURF1* (Supplementary Fig. 3b), *TRIB3* (Supplementary Fig. 4a), and *CBS* (Supplementary Fig. 4b). Each example is shown in five panels from top to bottom: (1) a LocusZoom plot of association within 1 Mb of the transcription start site (TSS) of the gene color by r^2 (LD) to the lead eQTL marker; y-axis, –log10(nominal p-value); x-axis, GRCh38 position; the dashed line marks the gene-specific nominal p-value significance threshold; (2) a Locuszoom plot as in (1) colored by callset, with the lead marker of each callset annotated; (3) gene annotations; (4) normalized gene expression versus the lead-marker genotype across 430 AFGR samples. Edge depth genotype is called from fitting a constrained Gaussian mixture model (GMM) from genomeSTRiP to continuous edge depth dosage. Boxes show the median and interquartile range (25th - 75th percentile). Individual samples are shown as points; and (5) a Bandage plot of the pangenome graph structure surrounding the lead variant in the pangenome graph. Reference nodes are colored in light blue and non-reference nodes in dark blue, with the lead edge indicated by an arrow. The dotted line in Supplementary Fig. 3a represents the inversion.

bioRxiv preprint doi: https://doi.org/10.64898/2026.07.01.735728; this version posted July 3, 2026. The copyright holder for this preprint (which was not certified by peer review) is the author/funder, who has granted bioRxiv a license to display the preprint in perpetuity. It is made available under a CC-BY 4.0 International license.

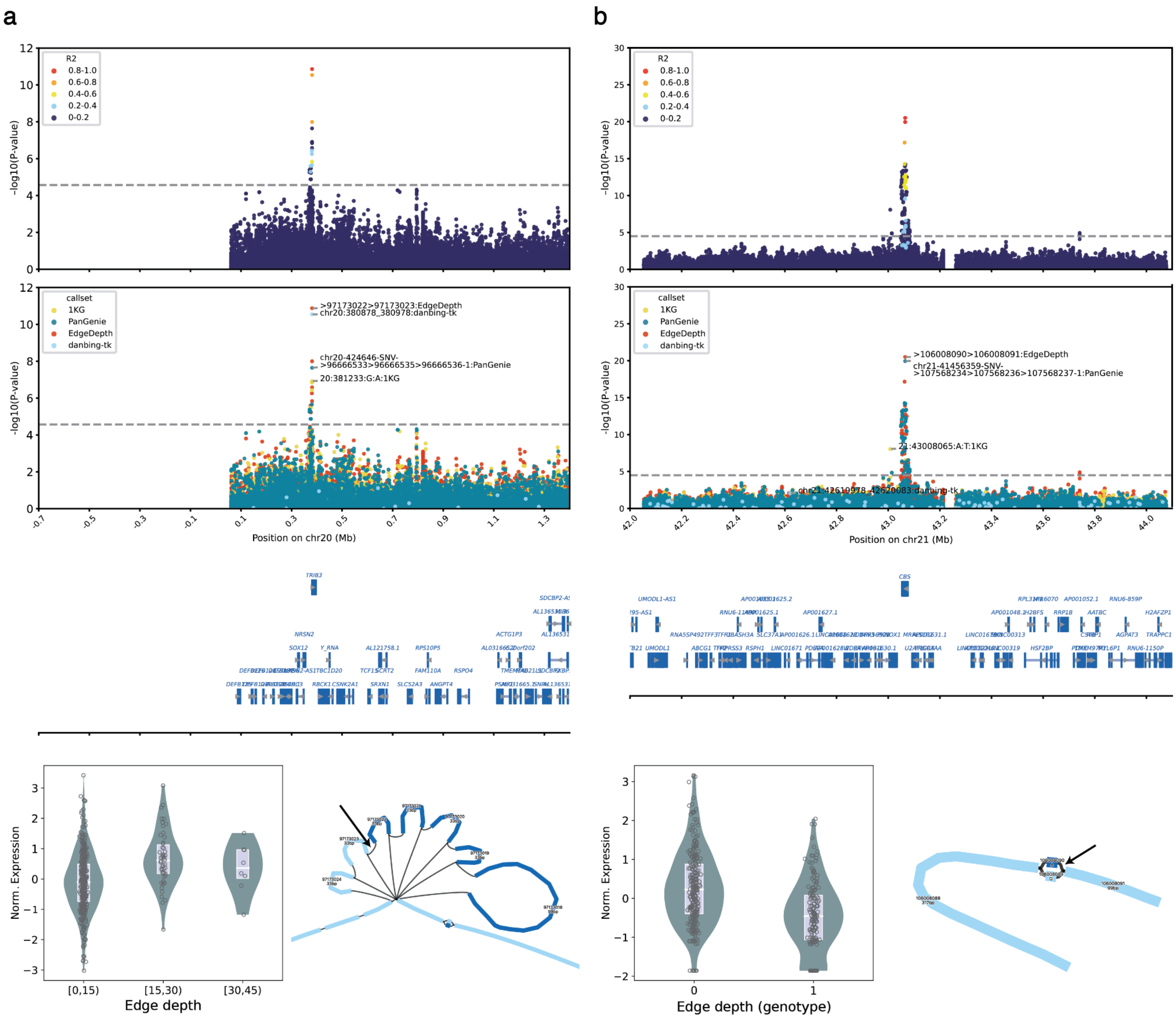

Supplementary Figure 4. See the legend for Supplementary Figure 3.

bioRxiv preprint doi: https://doi.org/10.64898/2026.07.01.735728; this version posted July 3, 2026. The copyright holder for this preprint (which was not certified by peer review) is the author/funder, who has granted bioRxiv a license to display the preprint in perpetuity. It is made available under a CC-BY 4.0 International license.

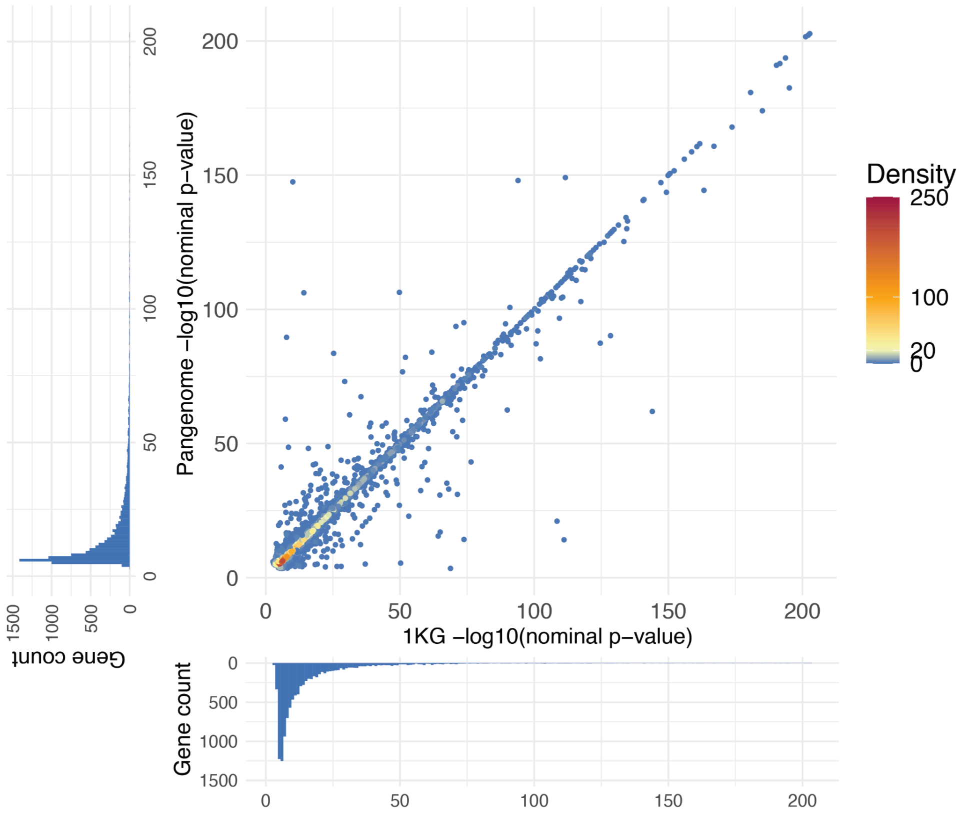

Supplementary Figure 5. Comparison of eQTL significance in pangenome callsets versus 1KG. Scatter plot comparing the strongest eQTL association signal from pangenome callsets with the strongest signal from the 1KG callset for each eGene in the joint eQTL analysis (N=10,223). For each point (eGene), the y axis shows the strongest -log10(nominal p-value) among variants from pangenome callsets, and the x axis shows the strongest -log10(nominal p-value) among variants from the 1KG callset. Points are colored by local density. Marginal histograms show the distributions of -log10(nominal p-value) for each axis.

bioRxiv preprint doi: https://doi.org/10.64898/2026.07.01.735728; this version posted July 3, 2026. The copyright holder for this preprint (which was not certified by peer review) is the author/funder, who has granted bioRxiv a license to display the preprint in perpetuity. It is made available under a CC-BY 4.0 International license.

a

	1KG vs. EdgeDepth	1KG vs. PanGenie	PanGenie vs. EdgeDepth
1KG	5,523	5,103	
PanGenie		5,246	5,433
EdgeDepth	4,874		4,883
Total eGenes	10,397	10,349	10,316
No. variants in each callset	17,119,745	15,720,533	15,968,535

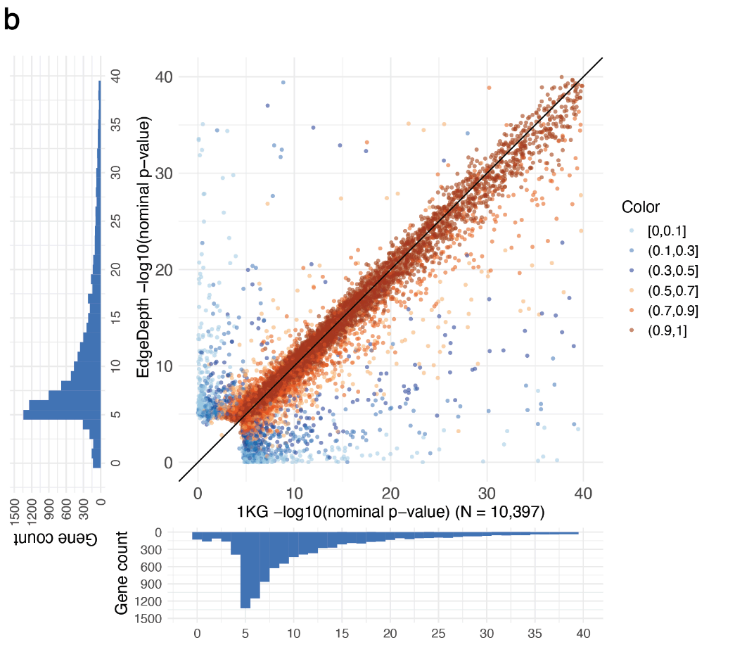

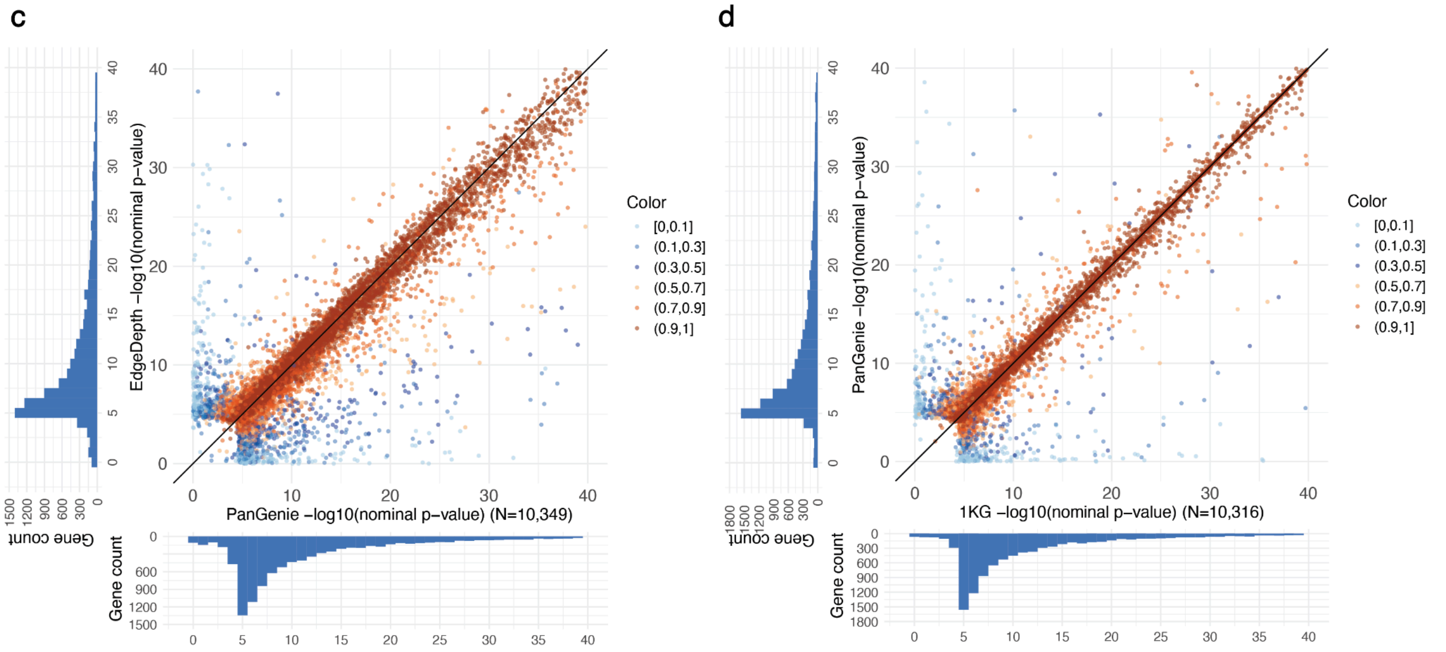

Supplementary Figure 6. Head-to-head comparison of eQTL significance at shared variants. Pairwise comparisons were performed among the 1KG, PanGenie, and EdgeDepth callsets using variants shared between each pair of callsets. A joint cis-eQTL analysis was performed for each pairwise comparison: 1KG vs. PanGenie, 1KG vs. EdgeDepth, and PanGenie vs. EdgeDepth. **(a)** Summary table showing, for each pairwise comparison, the number of eGenes contributed by each callset, the total number of eGenes identified, and the number of shared variants included in the analysis. **(b-d)** Scatter plots comparing eQTL association signals between each pair of callsets, zoomed to the range 0-40 on both axes. Each point represents the -log10(nominal p-value) of a lead variant from one callset and its corresponding matched variant in the other callset. Points are colored by the correlation between the lead variant and its matched variant. Marginal histograms show the distributions of -log10(nominal p-value) for each axis.

bioRxiv preprint doi: https://doi.org/10.64898/2026.07.01.735728; this version posted July 3, 2026. The copyright holder for this preprint (which was not certified by peer review) is the author/funder, who has granted bioRxiv a license to display the preprint in perpetuity. It is made available under a CC-BY 4.0 International license.

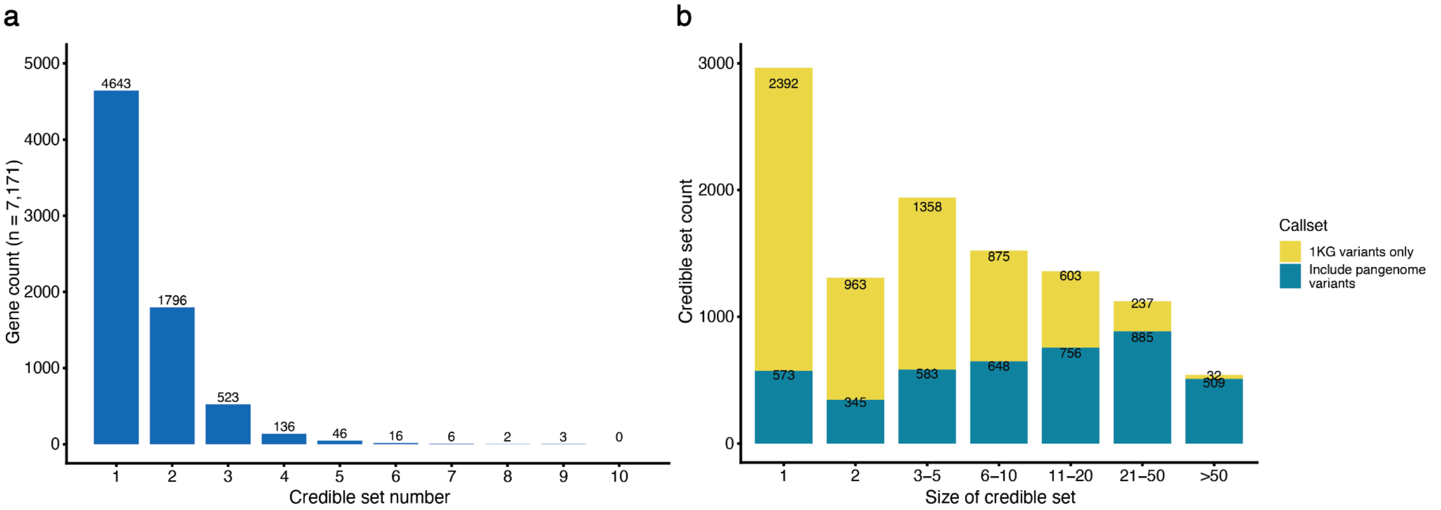

Supplementary Figure 7. Summary of credible sets (CSs) at fine-mapped eQTL signals from the merged HPRC2+1KG callset. (a) Histogram showing the number of credible sets per eGene (N = 7,171). **(b)** Histogram showing the number of variants per credible set. Each bar is colored to indicate how many credible sets of that size range include pangenome-specific variants or 1KG variants only.

bioRxiv preprint doi: https://doi.org/10.64898/2026.07.01.735728; this version posted July 3, 2026. The copyright holder for this preprint (which was not certified by peer review) is the author/funder, who has granted bioRxiv a license to display the preprint in perpetuity. It is made available under a CC-BY 4.0 International license.

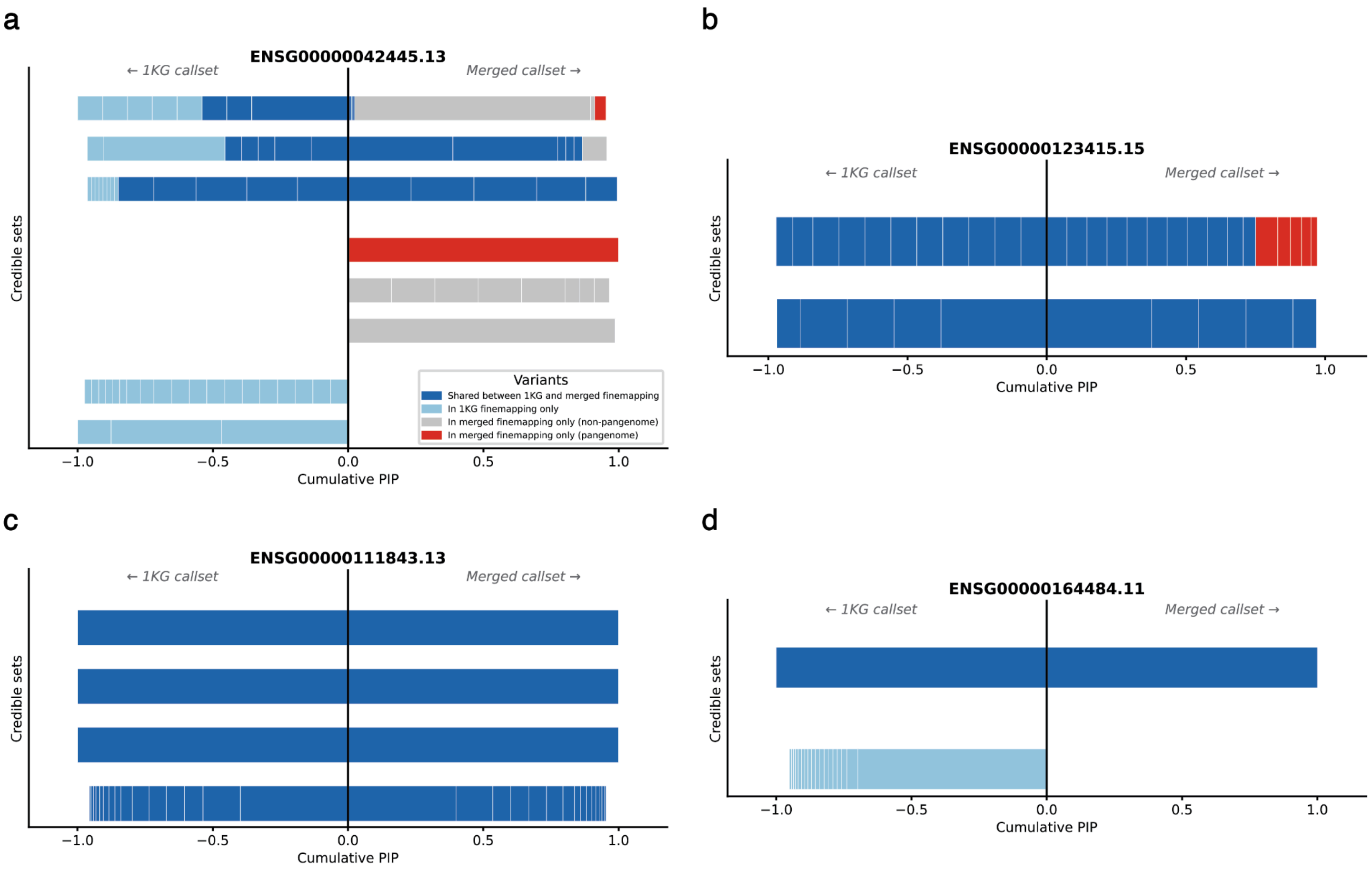

Supplementary Figure 8. Example fine-mapping results showing effects of pangenome-specific variants. Four representative examples illustrating categories of eGenes according to how posterior inclusion probabilities (PIPs) shift after adding pangenome-specific variants to 1KG for fine-mapping. Categories include pangenome refined signals, pangenome expanded signals, stable signals, and lost signals. **(a)** Example of "Pangenome refined" signals, defined as eGenes with at least one credible set (CS) dominated by pangenome-specific variants (summed pangenome variant PIP > 0.5), indicating that pangenome variants markedly improved signal discovery. Comparison of credible sets identified by fine-mapping using the 1KG callset alone (left) and the merged HPRC2+1KG callset (right). Each row represents one credible set (CS). Aligned rows indicate matched CSs, defined as CSs that share at least one variant across the two fine-mapping runs, whereas unaligned rows indicate CSs without a match in the other run. Variants within each CS are represented by rectangles and colored by matched status across the two runs and pangenome-specific variants. **(b)** Example of "pangenome expanded" signals, defined as eGenes where no CS is dominated by pangenome variants at least one CS in which pangenome variants contributed moderately (0.1 < summed pangenome variant PIP ≤ 0.5), and that CS matched a CS from fine-mapping with 1KG alone, indicating pangenome variants added additional candidates to the signal. **(c)** Example of stable signals, defined as eGenes for which all CSs were matched across both runs and pangenome variants did not contribute meaningfully or not at all (summed pangenome PIP ≤ 0.1), indicating fine-mapping was unaffected by the addition of pangenome-specific variants. **(d)** Example of lost signals, defined as eGenes with no pangenome-refined or pangenome-expanded credible sets, but with at least one CS present in the 1KG alone run that had no matched CS in the merged callset run, indicating that inclusion of pangenome-specific variants diffused previously identified signals.

bioRxiv preprint doi: https://doi.org/10.64898/2026.07.01.735728; this version posted July 3, 2026. The copyright holder for this preprint (which was not certified by peer review) is the author/funder, who has granted bioRxiv a license to display the preprint in perpetuity. It is made available under a CC-BY 4.0 International license.

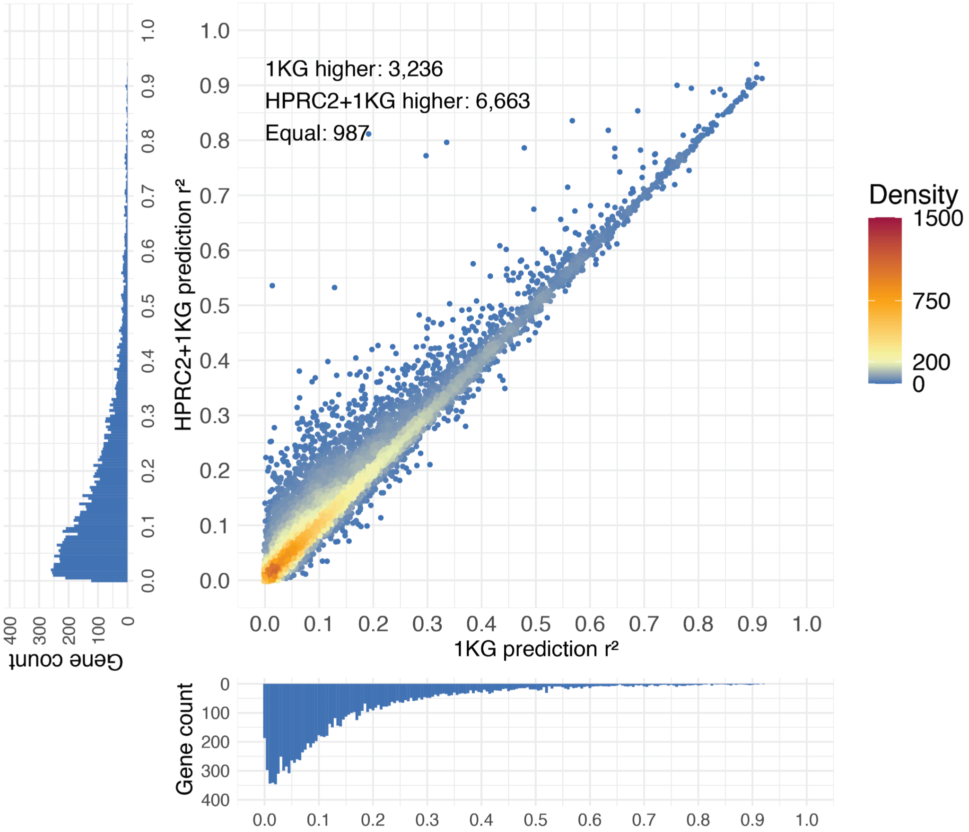

Supplementary Figure 9. Comparison of gene expression prediction accuracy between the HPRC2+1KG and 1KG callsets. Scatter plot comparing gene expression prediction accuracy for the union of eGenes discovered in eQTL analyses using the 1KG callset or the merged HPRC2+1KG callset (N=10,886). Prediction accuracy was quantified by the 5-fold cross-validated r^2 between predicted and observed expression, taking the highest adjusted r^2 across five methods (Lasso, ridge, elastic net, top-1 single best variant, and SuSiE) for each gene within each callset. For each point (eGene), the x axis shows the r^2 from the 1KG callset, and the y axis shows the r^2 from the merged HPRC2+1KG callset. Points are colored by local density. Marginal histograms show the distributions of r^2 for each axis.

bioRxiv preprint doi: https://doi.org/10.64898/2026.07.01.735728; this version posted July 3, 2026. The copyright holder for this preprint (which was not certified by peer review) is the author/funder, who has granted bioRxiv a license to display the preprint in perpetuity. It is made available under a CC-BY 4.0 International license.

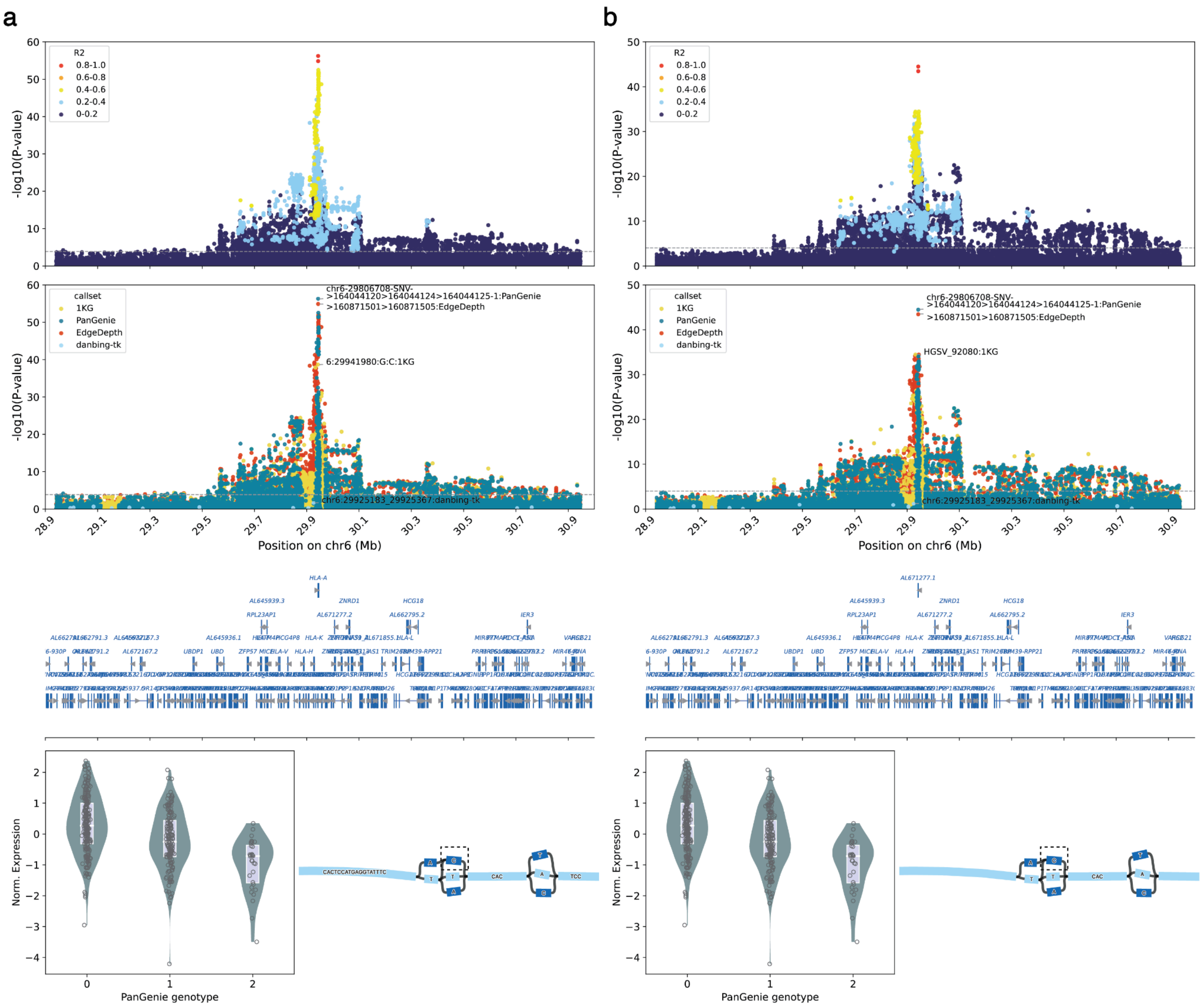

Supplementary Figure 10-14. Nine loci showing colocalization of GWAS and eQTL signals. These 9 loci are in addition to the *GBAP1* example shown in Fig. 5, namely *HLA-A* (Supplementary Fig. 10a), *HCG4P5* (Supplementary Fig. 10b), *CREM* (Supplementary Fig. 11a), *EHMT2* (Supplementary Fig. 11b), *UBE2R2* (Supplementary Fig. 12a), *PPM1G* (Supplementary Fig. 12b), *HSD17B8* (Supplementary Fig. 13a), *HCP5* (Supplementary Fig. 13b), and *HMG20A* (Supplementary Fig. 14). Each locus is shown in five panels from top to bottom (as in Supplementary Fig. 3): (1) a LocusZoom plot of association within 1 Mb of the transcription start site (TSS) of the gene color by r^2 (LD) to the lead eQTL marker; y-axis, –log10(nominal p-value); x-axis, GRCh38 position; the dashed line marks the gene-specific nominal p-value significance threshold; (2) a LocusZoom plot as in (1) colored by callset, with the lead marker of each callset annotated; (3) gene annotations; (4) normalized gene expression versus the lead-marker genotype across 430 AFGR samples. Edge depth genotype is called from fitting a constrained Gaussian mixture model (GMM) from genomeSTRiP to continuous edge depth dosage. Boxes show the median and interquartile range (25th - 75th percentile). Individual samples are shown as points; and (5) a Bandage plot of the pangenome graph structure surrounding the lead variant in the pangenome graph. Reference nodes are colored in light blue and non-reference nodes in dark blue. The lead variant is indicated by an arrow if it is an EdgeDepth variant or by a dashed box if it is a PanGenie variant.

bioRxiv preprint doi: https://doi.org/10.64898/2026.07.01.735728; this version posted July 3, 2026. The copyright holder for this preprint (which was not certified by peer review) is the author/funder, who has granted bioRxiv a license to display the preprint in perpetuity. It is made available under a CC-BY 4.0 International license.

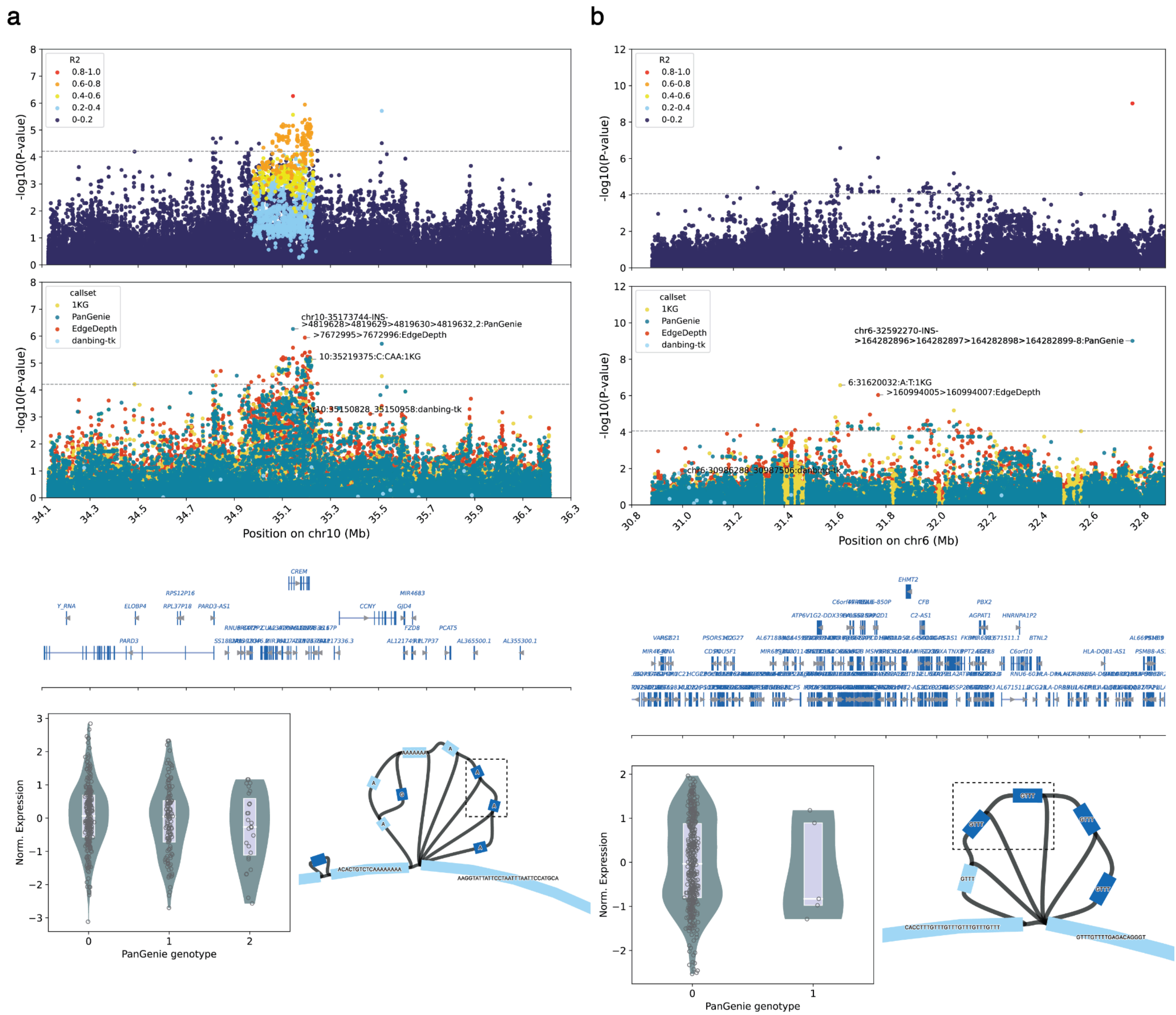

Supplementary Figure 11. See the legend for Supplementary Figure 10.

bioRxiv preprint doi: https://doi.org/10.64898/2026.07.01.735728; this version posted July 3, 2026. The copyright holder for this preprint (which was not certified by peer review) is the author/funder, who has granted bioRxiv a license to display the preprint in perpetuity. It is made available under a CC-BY 4.0 International license.

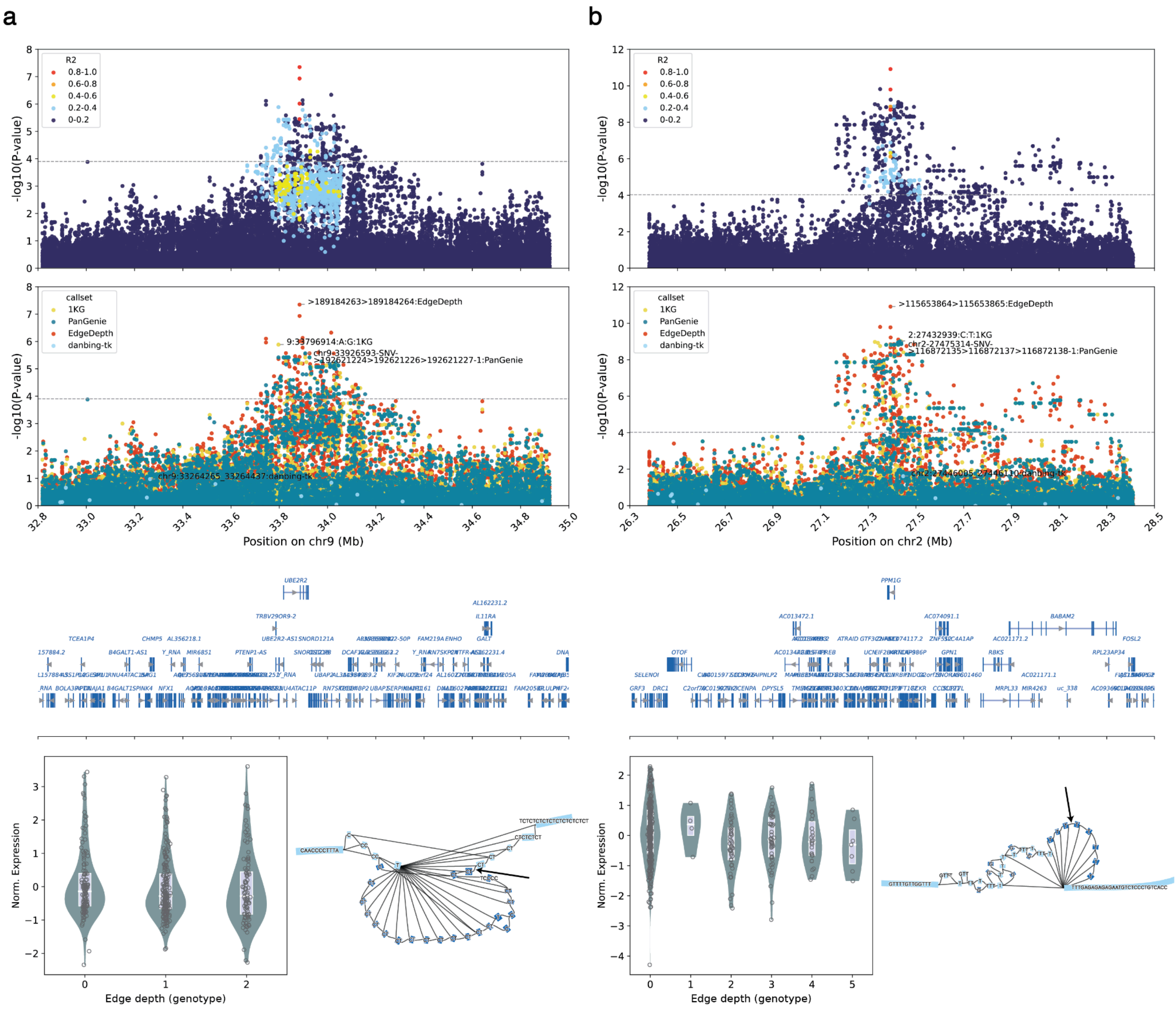

Supplementary Figure 12. See the legend for Supplementary Figure 10.

bioRxiv preprint doi: https://doi.org/10.64898/2026.07.01.735728; this version posted July 3, 2026. The copyright holder for this preprint (which was not certified by peer review) is the author/funder, who has granted bioRxiv a license to display the preprint in perpetuity. It is made available under a CC-BY 4.0 International license.

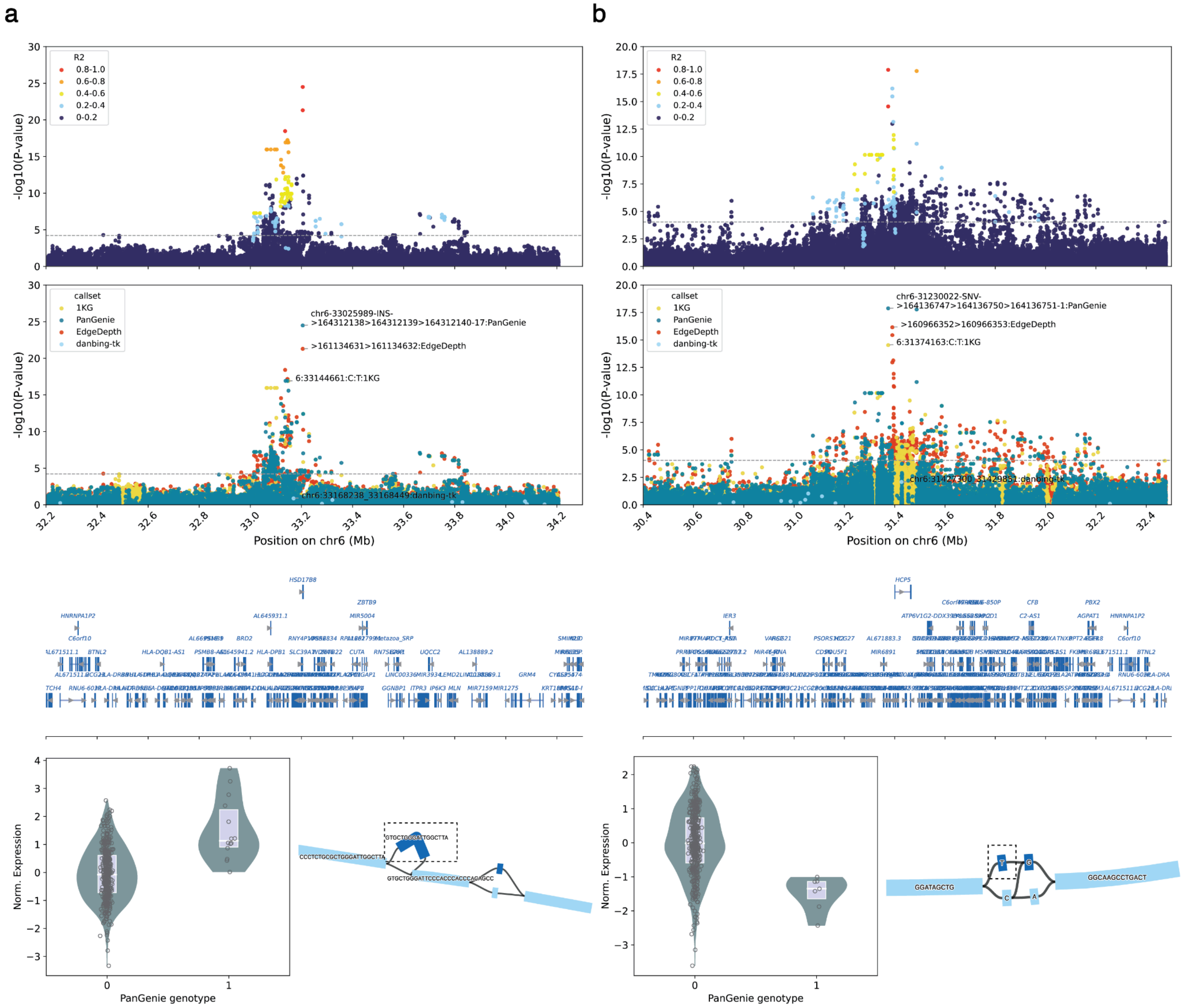

Supplementary Figure 13. See the legend for Supplementary Figure 10.

bioRxiv preprint doi: https://doi.org/10.64898/2026.07.01.735728; this version posted July 3, 2026. The copyright holder for this preprint (which was not certified by peer review) is the author/funder, who has granted bioRxiv a license to display the preprint in perpetuity. It is made available under a CC-BY 4.0 International license.

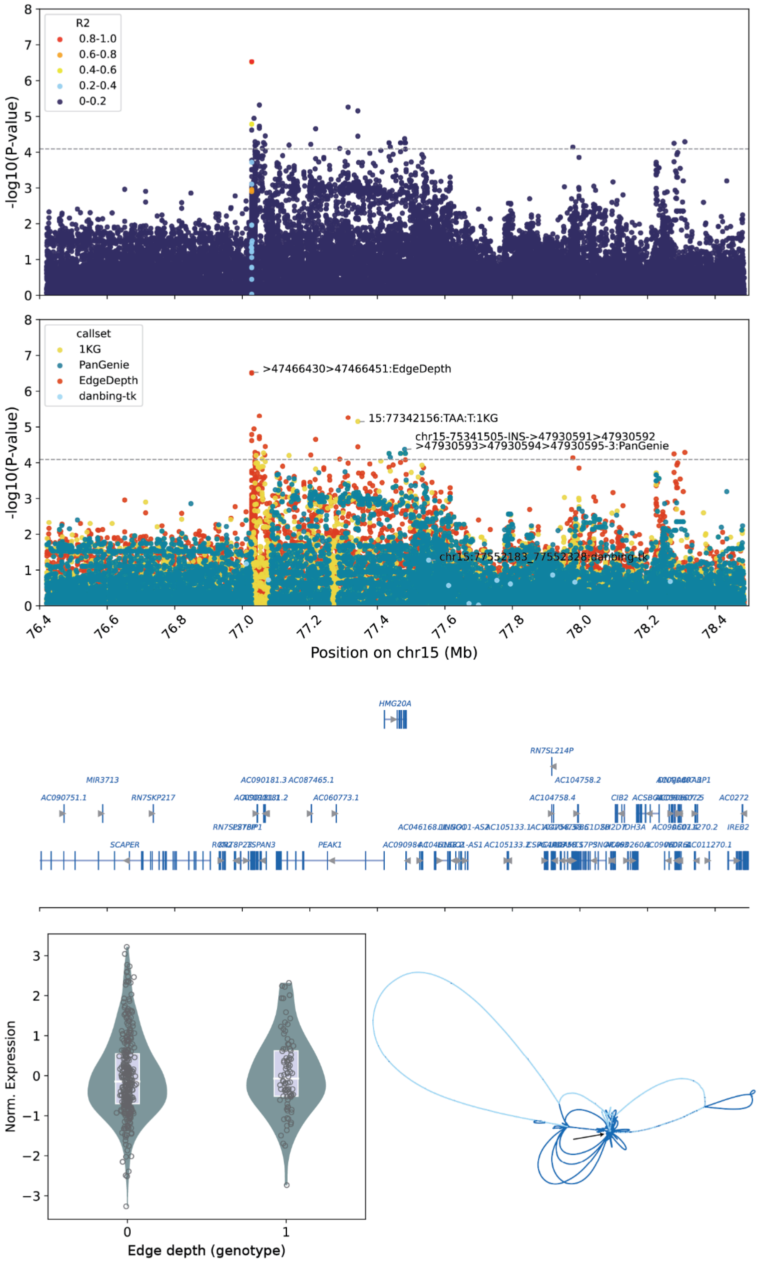

Supplementary Figure 14. See the legend for Supplementary Figure 10.

bioRxiv preprint doi: https://doi.org/10.64898/2026.07.01.735728; this version posted July 3, 2026. The copyright holder for this preprint (which was not certified by peer review) is the author/funder, who has granted bioRxiv a license to display the preprint in perpetuity. It is made available under a CC-BY 4.0 International license.

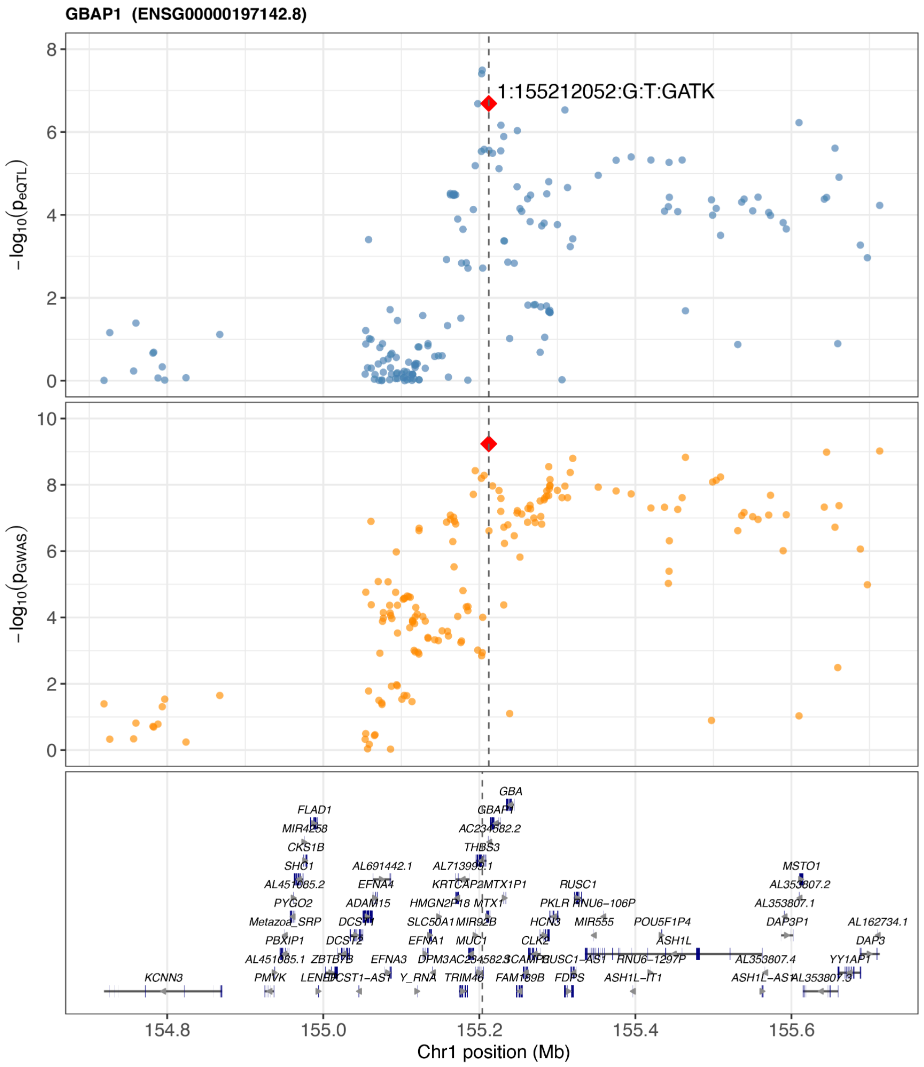

Supplementary figure 15. GWAS and eQTL colocalization at the *GBAP1* locus. LocusZoom plot of the *GBAP1* locus showing 1KG variants shared between the eQTL and GWAS analysis within 500 kb of the lead eQTL marker. The top panel shows the eQTL signal at *GBAP1*. The middle panel shows the Crohn's disease GWAS signal. The bottom panel shows gene annotations in the window. The red diamond marks the variant proposed as the shared causal variant by coloc[31].

Supplementary File 1. Study information for the 41 GWAS used in the GWAS and eQTL colocalization analysis, including study ID, trait, sample size, ancestry, and publication.

Supplementary File 2. Summary of the 10 GWAS and eQTL colocalization loci, reporting the eQTL lead variant, eGene, colocalization posterior probability (H4) and GWAS trait at each locus.
